# The Origin of a Coastal Indigenous Horse Breed in China Revealed by Genome-Wide SNP Data

**DOI:** 10.3390/genes10030241

**Published:** 2019-03-21

**Authors:** Hongying Ma, Sheng Wang, Guorong Zeng, Jintu Guo, Minghao Guo, Xianggui Dong, Guoying Hua, Yu Liu, Min Wang, Yao Ling, Xiangdong Ding, Chunjiang Zhao, Changxin Wu

**Affiliations:** 1 College of Animal Science and Technology, China Agricultural University, Beijing 100193, China; B1303258@cau.edu.cn (H.M.); Shengwang@cau.edu.cn (S.W.); xgdong@cau.edu.cn (X.D.); hgyyxg@cau.edu.cn (G.H.); yliu316@cau.edu.cn (Y.L.); haohe@cau.edu.cn (M.W.); lingzi@cau.edu.cn (Y.L.); chxwu@cau.edu.cn (C.W.); 2 Equine Center, China Agricultural University, Beijing 100193, China; 3 National Engineering Laboratory for Animal Breeding, Beijing 100193, China; 4 Key Laboratory of Animal Genetics, Breeding and Reproduction, Ministry of Agriculture, Beijing 100193, China; 5 Jinjiang Animal Husbandry and Veterinary Station, Fujian 362200, China; 13655922266@163.com (G.Z.); m15764251673@163.com (J.G.); guominghao_1325@163.com (M.G.); 6 Beijing Key Laboratory for Genetic Improvement of Livestock and Poultry, Beijing 100193, China

**Keywords:** Jinjiang horse, origin, ancestry, Single Nucleotide Polymorphism array

## Abstract

The Jinjiang horse is a unique Chinese indigenous horse breed distributed in the southern coastal areas, but the ancestry of Jinjiang horses is not well understood. Here, we used Equine SNP70 Bead Array technology to genotype 301 horses representing 10 Chinese indigenous horse breeds, and we integrated the published genotyped data of 352 individuals from 14 foreign horse breeds to study the relationships between Jinjiang horses and horse breeds from around the world. Principal component analysis (PCA), linkage disequilibrium (LD), runs of homozygosity (ROH) analysis, and ancestry estimating methods were conducted to study the population relationships and the ancestral sources and genetic structure of Jinjiang horses. The results showed that there is no close relationship between foreign horse breeds and Jinjiang horses, and Jinjiang horses shared a similar genetic background with Baise horses. TreeMix analysis revealed that there was gene flow from Chakouyi horses to Jinjiang horses. The ancestry analysis showed that Baise horses and Chakouyi horses are the most closely related ancestors of Jinjiang horses. In conclusion, our results showed that Jinjiang horses have a native origin and that Baise horses and Chakouyi horses were key ancestral sources of Jinjiang horses. The study also suggested that ancient trade activities and the migration of human beings had important effects on indigenous horse breeds in China.

## 1. Introduction

There are over five million horses in China, the majority of which are indigenous horses that are distributed widely across the rural areas of China. The largest horse populations exist in the northern and southwestern provinces [1]. The Jinjiang horse is the only Chinese indigenous horse breed (CHB) located in the southeast coastal areas of Fujian Province, and it is named for the prefecture of the river and the city where Jinjiang horses are mainly distributed. Most Jinjiang horses have chestnut or bay coat colors, while a few show buckskin, black, or pinto colorations. The various coat colors stem from their breeding activities, in which stallions and mares were not intensively selected and for whom there are no systematic and well-designed breeding strategies. Unlike other horse breeds in South China, which inhabit the relatively cool and dry mountainous southwest provinces, Jinjiang horses exhibit specific adaptations to the hot and humid conditions of the south coastal areas [2]. Moreover, Jinjiang horses have muscular bodies and draft-type conformations, whereas other southern horse breeds are usually slim and dwarf. Thus, the Jinjiang horse, as a unique breed, is significantly different from other horse breeds in South China in both its habitat and its external appearance [3].

However, the origin of Jinjiang horses remains unknown due to a lack of historical records. There no longer exist indigenous horse breeds in the provinces adjacent to Fujian Province, namely, the Zhejiang Province, Jiangxi Province, and Guangdong Province, which make the ancestry of Jinjiang horses more elusive. Because Quanzhou, where Jinjiang horses are distributed, was the most important and pivotal port of the Marine Silk Road (AD 12 to AD 14) [4], this ancient port city made maritime trade flourish along the coastal areas of South China and reached to the Arab world and beyond. Thus, it was speculated that the origin of the Jinjiang horse may be related to that of Arabian horses. Due to the popularization of mechanization in agriculture, Jinjiang horses are now at the edge of extinction [5]. A study of the origin of Jinjiang horses will provide meaningful information in support of the conservation and utilization of Jinjiang horses. Despite cultural and scientific interest in Jinjiang horses, few efforts a made to better understand the origin of this unique CHB.

Thus far, most phylogenetic studies on horse populations have been based on mitochondrial data [6,7,8,9,10,11,12], microsatellite sites [13,14,15,16], or Y-chromosome data [17,18,19]. Although much progress was achieved through such studies, obvious limitations remain. Studies based on Y-chromosome markers or mitochondrial DNA (mtDNA) could only show the information of the patrilineal or maternal lines of horses. Also, a limited number of polymorphic microsatellite sites were applied in phylogenetic studies, which could not result in high resolution of the populations. Genome-wide high-density single nucleotide polymorphism (SNP) arrays, based on whole genome sequencing, have the advantage of mass information. Therefore, they are being increasingly applied in the research areas of origin and evolution [20,21,22,23]. In recent years, genome-wide high-density SNP arrays was used in several genetic studies to analyze the origins of horses, and the genetic relationships among horse populations was revealed, providing insight into the origins of those horse breeds [24,25,26]. Although genome-wide SNP arrays provide a high density of polymorphic sites at the genomic level for phylogenetic study, their main limitation is that they only reveal part of the whole SNP pool of the populations under investigation. Because the SNP sites of the arrays are selected by screening across a limited number of breeds, only shared polymorphisms can be detected when the arrays are used to genotype other breeds, and some unique SNPs of the other populations cannot be detected when breed-specific SNP sites are not included in the commercial arrays. Whole genome resequencing, with decreasing costs, will overcome the above limitation. Overall, however, the genome-wide high-density SNP arrays provide a powerful tool for horse phylogenetic studies.

In the present study, the genome-wide high-density SNP arrays were employed to genotype horses from 10 Chinese indigenous breeds representing the five horse breed groups of China, which were classified according to the geographic distributions and genetic relations of the indigenous breeds, namely, the Mongolia Group, the Hequ Group, the Tibet Group, the Kazakh Group, and the Southwest Group. Additionally, the genotyped genomic SNPs data of 14 foreign horse breeds from previous studies [25] were also integrated to analyze the genetic structure of Jinjiang horses and to explore the genetic relationship between Jinjiang horses and the domestic or overseas breeds to reveal the origin of Jinjiang horses.

## 2. Materials and Methods

### 2.1. Sample Collection and DNA Extraction

Blood samples of 301 horses from 10 CHBs and five donkeys were collected. The samples were obtained following the principles approved by the Animal Care and Use Committee of China Agricultural University (Permit Number: XK257). Genomic DNA was isolated by standard phenol/chloroform extraction methods.

**Figure 1:**
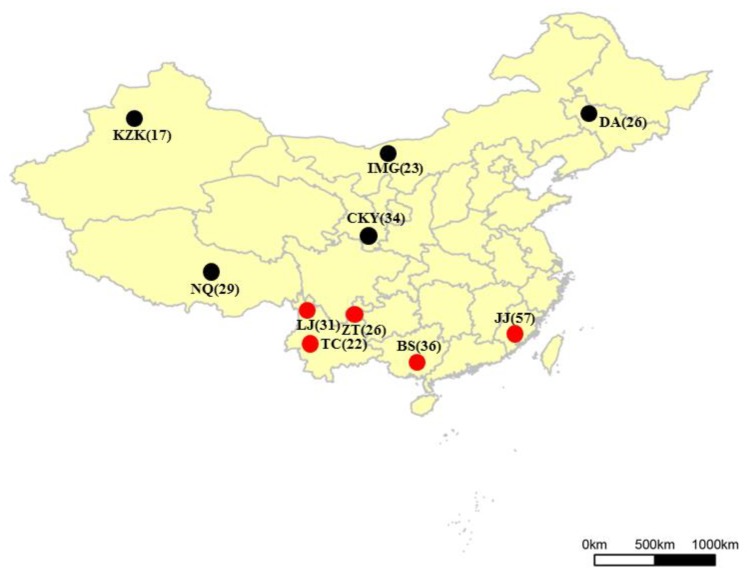
Geographic distribution of the Chinese horses studied. IMG, Mongolian horse; DA, Daan horse; CKY, Chakouyi horse; NQ, Naqu horse; KZK, Kazakh Horse; BS, Baise horse; JJ, Jinjiang horse; TC, Tengchong horse; ZT, Zhaotong horse; LJ, Lijiang horse. Red dots indicate the breeds in South China; black dots, the horses in North China. (The map was configured using R Packages).

The sampled horse breeds included the Mongolian horse (IMG) from Inner Mongolia, representing Mongolia breed group, the Daan horse (DA) in Jilin Province (belonging to the Mongolia horse group), the Chakouyi horse (CKY) in Gansu Province (representing the Hequ breed group), the Naqu horse (NQ) in Tibet (standing in for the Tibet breed group), the Kazakh horse (KZK) in Xinjiang (representing the Kazakh breed group), and horse breeds from South China (mainly belonging to the Southwest breed group, which consisted of the Baise horse (BS) in Guangxi Province, the Tengchong horse (TC), Zhaotong horse (ZT), and Lijiang horse (LJ) in Yunnan Province, and the Jinjiang horse (JJ) in Fujian Province, which was the only breed from Southeast China (Figure 1)). Five donkeys from Dezhou in Shandong Province were also sampled. Detailed information is shown in Table S1. Genotyping data series in this study have been stores in the NCBI repository, with the accession code GSE128376.

### 2.2. Datasets

The dataset of worldwide horse populations consisted of the data collected in this study and previously published data, comprising a total of 653 animals from 24 horse breeds. The sampled horses of the 10 Chinese indigenous breeds in the present study were genotyped with the Illumina Equine SNP70 Bead Array. The previously published data of horses from 14 western breeds, genotyped with Illumina Equine SNP50 Bead Array, were from a study of Petersen et al. [25]. There are 45,703 markers shared by the SNP70 and SNP50 arrays. Quality control of the raw data was carried out using PLINK v1.07 [27]. The following SNPs were discarded: (1) SNPs with Hardy-Weinberg Equilibrium *p*-value < 1 × 10^−5^; (2) SNPs that were missing more than 10% of their genotype data; and (3) SNPs with a minor allele frequency less than 1%. Individuals with more than 10% missing genotyped data were also removed. The total SNPs left in the merged dataset of the worldwide horse populations were 39,187, while there were 57,597 filtered SNPs in the thinned data of the 10 Chinese indigenous breeds (Tables S1 and S2).

### 2.3. Population Divergence

To better infer the genetic structure of the Jinjiang horses and the 23 other worldwide horse breeds, we constructed a phylogenetic tree using the genome-wide high-density SNP data according to the following steps. First, the identical by state (IBS) distance matrix between individuals was generated by PLINK v1.07 [27] using the resulting 39,187 SNP sites. Second, based on the distance matrix, the neighbor joining (NJ) tree was constructed by MEGA v6 [28] and displayed by FigTree v1.4.0 [29]. For this analysis, we used donkey samples as an outgroup. We also performed principal components analysis (PCA) with the filtered 39,187 SNPs using the GCTA software (v1.24.2) [30]. The genetic relationship matrix and the covariance matrix were inferred from the PLINK format files (.ped and .map) with the parameters “-make -grm -pca 3.” Then, the eigenvectors were defined based on the inferred covariance matrix, and the PCA biplot was plotted with ggplot2 (R Packages). The construction of the population structure was analyzed with the program Admixture v1.3 [31]. This program estimated the admixture proportions among the 24 horse breeds with the 39,187 SNPs. Nineteen scenarios (ranging from K = 2 to 20, Appendix A) were selected for genetic clustering with the parameters “major convergence criterion = 0.01”. A cross-validation approach was used to determine the most likely number of populations (K) in the data. Additionally, we estimated the relationships between the Jinjiang horses and other 23 horse breeds in the dataset using TreeMix v1.13 [32] to determine the historical relationships between these breeds in terms of splits and migrations (mixtures). We used donkeys as the root population of the TreeMix analysis and set the SNP block size parameter to 10. TreeMix was run iteratively for values of the migration parameter (-m) between 1 and 8 (Appendix B). The f index, representing the fraction of the variance in the sample covariance matrix (Ŵ) calculated by the model covariance matrix (W), was used to identify the number of modeled migration events that best fit the data [32]. 

To study the relationship between the Jinjiang horse and the other CHBs, the 57,597 filtered SNPs were applied to conduct the subsequent analyses. A phylogenetic tree merely containing the Chinese horses was constructed with the same steps mentioned above. An analysis of population structure was carried out using the software Admixture v1.3 [31], which uses a model-based estimation of individual ancestry for a range of prior values of K defined by the user. The values of K were in the range from 2 to 12 (Appendix C) for accommodating a potential population structure within the breeds. The most suitable value of K was determined by the cross-validation approach. A principal components analysis was carried out using the GCTA software [30] with the same settings described above. A maximum likelihood tree of the 10 Chinese breeds was constructed using TreeMix v1.13 [32] based on the genome-wide allele frequency data. The TreeMix was run iteratively for values of the migration parameter from 1 to 5 (Appendix D), using the f index to identify the most suitable migration event model.

### 2.4. LD Decay

Linkage disequilibrium (LD) levels for Chinese horse populations were assessed by the genotype correlation coefficient (*R*^2^) between any two loci (within and between different chromosomes) using PLINK v1.07 [27]. The parameters were set as “-blocks no -pheno -req -blocks -max -kb 10,000.” Then, visualizations of LD decays among horse populations across the whole genome were generated using R scripts.

### 2.5. Identical by Descent Analyses

The data of 301 horse individuals genotyped with the 57,597 SNPs at the whole-genome level served as input for the identical by descent (IBD) detection. The frequencies of shared haplotypes between Jinjiang horses and each of the other CHBs were estimated with per 10,000 bp bins using IBDLD software (v3.37) [33]. The parameters were set as “-plinkbf int evolution -method GIBDLD -ploci 10 -nthreads 24 -step 0 -hiddenstates 3 -segment –length 10 -min 0.8”. The calculation of normalized IBD (nIBD) between the Jinjiang horses and each of the other CHBs was as follows: nIBD = cIBD/tIBD, where cIBD = count of all haplotypes IBD between the Jinjiang horse and each of the other CHBs, and tIBD = total pairwise comparisons between the Jinjiang horse and each of the other CHBs. 

### 2.6. Runs of Homozygosity and Genetic Diversity Analysis

Runs of homozygosity segments (ROH) were determined with an overlapping window approach implemented in PLINK v1.07 [27]. The final segments were called ROH if the minimum length of the homozygous segment was greater than 500 kb and comprised more than 50 homozygous SNPs, whereas one heterozygote and two missing genotypes were permitted within each segment. The analysis was based on the following settings: minimum SNP density was set to one SNP per 500 kb with a maximum gap length of 1000 kb. Three ROH parameters, genome length covered by ROH(S_ROH_), number of ROH(N_ROH_), and autozygosity (F_ROH_) were computed.

Indices of genetic diversity, including observed heterozygosity (H_O_) and expected heterozygosity (H_E_), as well as the inbreeding coefficient (f), were also determined with PLINK v1.7 using the command --het.

### 2.7. Formal Test of Ancestor Admixture 

The genome-wide most likely ancestry of Jinjiang horses was estimated using the f4 Ratio Estimation method of the ADMIXTOOLS Software Package [34] with default parameters. Applying f4 ratio estimation, we investigated the ratio of f4 (A, O; X, C)/f4 (A, O; B, C). The population X is an admixture of populations B and C. We calculated this ratio using the Naqu horse as A, the Baise horse as B, the other CHBs as C, the Jinjiang horse as X, and the donkey as O.

### 2.8. Estimating the Ancestry Proportion of the Jinjiang Horse

To infer the potentially genetic proportion of ancestral sources of Jinjiang horses from other horses, we estimated the admixture proportion using the LD based admixture inference algorithm implemented in ALDER v1.03 [35]. Based on the allele frequency difference in ancestral populations, the algorithm computes SNP correlations in an admixed target population and weights the correlations. We used the Baise horse and the Chakouyi horse as the reference populations and the Jinjiang horse as the target population. We created the weighted LD curves for the tests with the parameters “mincount = 4, binsize = 0.0005, maxdis = 0.5, fast_snp_read = NO.”

## 3. Results

### 3.1. Genetic Relationships between the Jinjiang Horses and Worldwide Horse Breeds

**Figure 2:**
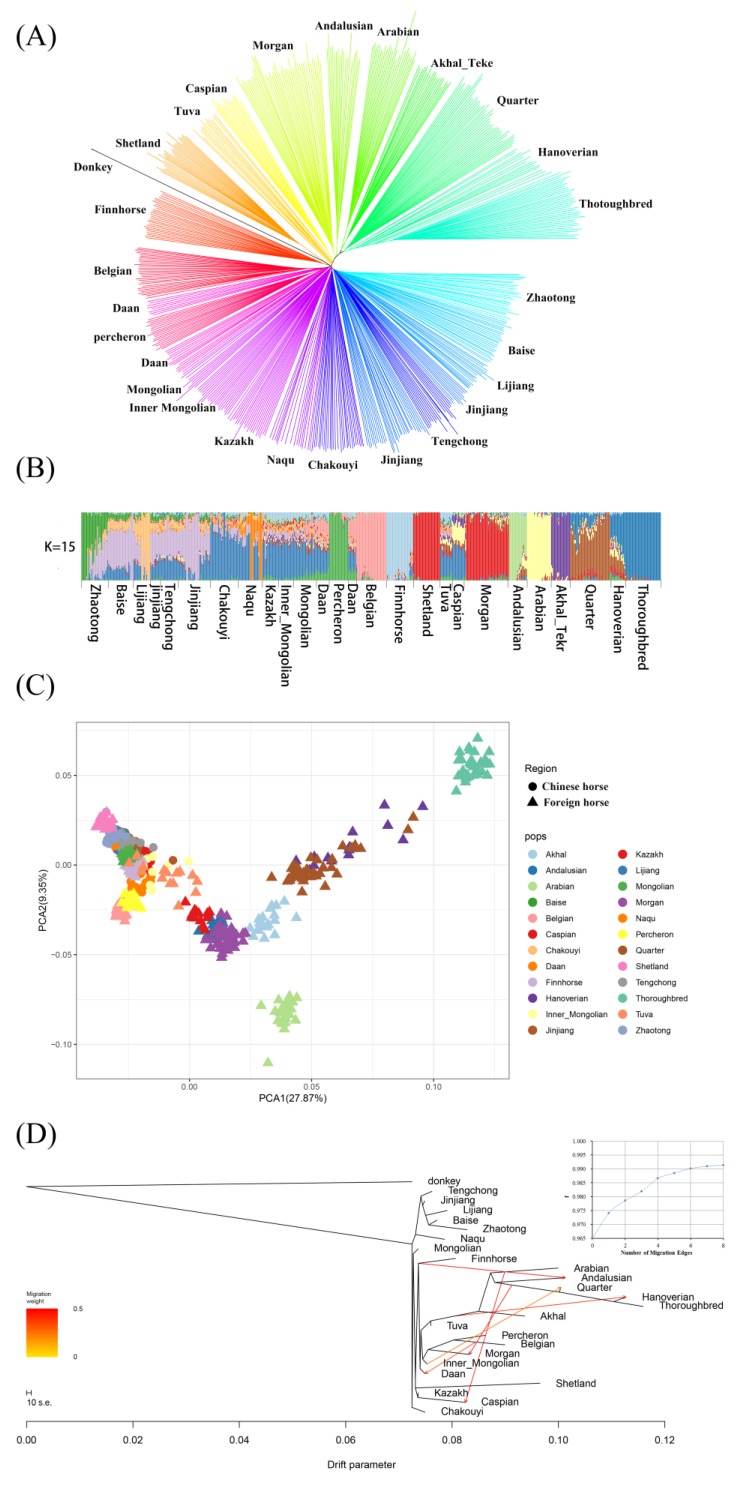
Genetic relationships among the Jinjiang horse and 23 other horse populations and their population structures. (**A**) Relationships among the Jinjiang horse and the worldwide horse populations, illustrated by a neighbor-joining tree. The populations studied are indicated with different colors and the names of the breeds. Five donkeys were used as an outgroup. The neighbor-joining tree was constructed with 580 individuals from 10 Chinese indigenous horse breeds and 14 foreign horse populations, using 39,187 filtered single nucleotide polymorphisms (SNPs) per sample. (**B**) ADMIXTURE analysis of the Jinjiang horse and the worldwide horse populations. Results with K = 15 hypothetical ancestral populations. Each column indicates an individual. The column group represents a horse population. (**C**) Principal components analysis for the Jinjiang horses and the worldwide horse populations. The Chinese indigenous horse breeds and foreign horse populations are represented with different symbols (circles and triangles, respectively). The populations studied are indicated with different colors. (**D**) Migrations detected among the foreign and Chinese horse breeds studied by the TreeMix program with six migration events.

The genotype data of the Chinese indigenous horses from the 10 breeds, which were generated in the present study, as well as that of the 14 breeds of foreign horses from the previous study were used in the phylogenic analysis. After quality control, a total of 580 individuals with 39,187 SNP positions were involved in the subsequent analyses (Table S1). A neighbor-joining tree was constructed using the worldwide horses with the filtered data (Figure 2A). Major clades of the tree showed breed groups, including the group of CHBs, the breeds which were recently admixed with or partly derived from Thoroughbreds (Quarter horse, Hanoverian horse), the Middle Eastern breeds (Akhal-Teke and Arabian horses), the group consisting of the Caspian horse and the Tuva horse, and the group containing the Shetland horses, Andalusian horse, and Morgan horse. The Belgian horse, Percheron horse, and Mongolian horse clustered together, which indicated that they have a close relationship, especially between the Daan horse and the Inner Mongolian horse. The Jinjiang horses were assigned into two clusters, one of which was close to the Chakouyi horses and another clustered with the Southwest breed group consisting of the Tengchong horses, the Lijiang horses, the Baise horses, and the Zhaotong horses.

Based on genetic co-ancestry analyses [36], we partitioned all individuals into known groups by varying the number of presumed ancestral populations (Appendix A, K ranging from 2 to 20). The cross validation (CV) statistics were used to choose the most suitable number of clusters, whose lowest value was K = 15 (Appendix A). When K was 15, the results showed that CHBs were derived from similar genetic backgrounds. The results revealed an evident admixture between the Daan horse and the draft horse breeds (i.e. the Belgian horse and the Percheron horse (Figure 2B)). The ternary principal components analysis (PCA) revealed similar results with those of the ancestry estimation, while also showing closer genetic relationships among the Chinese horse breeds and evidence that some breeds in North China are closely related to the heavy horse breeds (Figure 2C).

In addition to PCA and clustering analyses, the TreeMix algorithm was used to place the Jinjiang horse on a tree based on a maximum likelihood estimation approach. First, the relationships between the Jinjiang horse and other worldwide horse populations were estimated. The f index representing the fraction of the variance in the sample covariance matrix (Ŵ) was calculated with the model covariance matrix (W), as a function of modeling the number of modeled migration events. More migration edges did not seem to further increase the variance explained by the phylogenetic model, as the f index reached an asymptote above 6 migration edges (Figure 2D, inset), so m = 6 was chosen as optimum migration mode in this dataset. The results showed some well-known gene flows, such as that from Thoroughbreds to Morgan horses, and from Arabian horses to Caspian horses, and several other migration events, which mainly happened between foreign breeds. Notably, the migration from foreign draft horse breeds to the Daan horses was the only gene flow detected between Chinese and foreign horses. The results showed that there were no migration events between Jinjiang horses and overseas populations, including Arabian horses (Figure 2D).

### 3.2. Population Genetic Relationships between the Jinjiang Horse and Other Chinese Indigenous Horse Breeds

**Figure 3:**
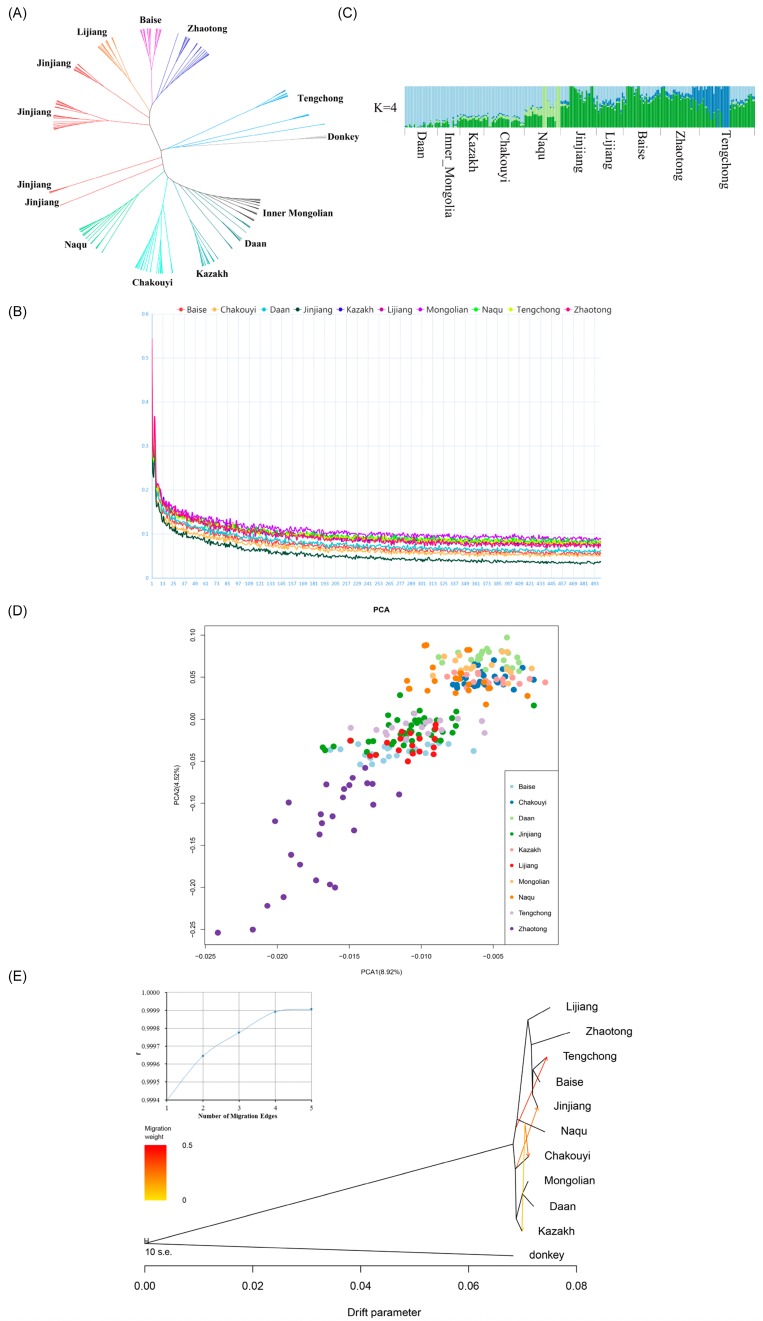
Genetic relationships between the Jinjiang horse and other Chinese indigenous horse populations and their population structures. (**A**) Relationships among the studied Chinese indigenous horse breeds (CHBs) illustrated by a neighbor-joining tree. The populations studied are indicated with different colors and the names of the breeds. Five donkeys were used as an outgroup. The neighbor-joining tree was constructed with 234 individuals from 10 Chinese indigenous breeds, using 57,597 SNPs having passed quality filtration per sample. (**B**) Linkage disequilibrium decay of the studied Chinese indigenous horse breeds. The studied populations are indicated with different colors. The analysis was conducted using 57,597 filtered SNPs per sample, with 234 individuals from 10 Chinese indigenous horse breeds. (**C**) ADMIXTURE analysis of the studied Chinese indigenous horse breeds. Results with K = 4 hypothetical ancestral populations. Each column indicates an individual. The column group represents a horse population. (**D**) Principal components analysis of the studied Chinese indigenous horses. The studied Chinese indigenous horse populations are indicated with different colors. (**E**) Migrations detected among the studied Chinese indigenous horse breeds by the TreeMix program with four migration events.

Because the Jinjiang horse is solely genetically related to other CHBs according to the results of the analyses of the worldwide horse data, the subsequent studies were focused on the 10 CHBs to investigate the genetic relationships between the Jinjiang horse and other Chinese horse breeds in the following analyses using the 57,597 SNPs that passed quality filtration. A neighbor joining (NJ) tree only containing Chinese indigenous horses showed that Jinjiang horses form four branches, with most Jinjiang horses following their geographical distributions and clustering with horse breeds from Southwest China, while a few individuals of the breed are relevant to horse breeds in North China (Figure 3A). 

Artificial selection tends to reduce genetic diversity, and commercial breeds have undergone stronger artificial selection than local breeds have. The impact of artificial selection could also be detected in the genome LD levels in each population, as artificial selection can facilitate the increase of LD within a population [37]. The result of LD analyses showed that Jinjiang horses have the lowest level of LD, which indicates that they have not been subjected to intensive selection (Figure 3B).

The results from both analysis of ADMIXTURE (K = 4, Figure 3C) and PCA (Figure 3D) showed that Jinjiang horses have closer relationships with horse breeds from South China, especially Baise horses. The results also indicated a differentiation between horse populations from South China and North China. A TreeMix analysis of the Chinese indigenous breeds was performed by setting the migration edges at 3, as there was no further increase of the variation explained when the value of migration edges was greater than 3 (Figure 3E, inset). The results showed that there was a significant gene flow from the Chakouyi horse to the Jinjiang horse. In addition, migration events from the Naqu horse to the Chakouyi horse, and from the Northern horses to the Zhaotong horses, were also observed in the results (Figure 3E). 

### 3.3. Runs of Homozygosity and the Genetic Diversity of the Worldwide Horse Breeds

F_ROH_ can quantify more recent inbreeding. In the horse populations studied, the highest F_ROH_ was observed in the Andalusian horses (mean F_ROH_ = 0.1022), followed by the Thoroughbreds (mean F_ROH_ = 0.0990). The lowest value was obtained for the Chakouyi horses (mean F_ROH_ = 0.0060), and the Naqu population also had very low F_ROH_ values. 

The observed heterozygosity of the horse breeds ranged from 0.2955 to 0.2748 (Table 1). The Morgan horses and Jinjiang horses showed relatively high heterozygosity, and the lowest value was observed in the Akhal Teke horse population. The expected heterozygosity of the Belgian horses and the Arabian horses was slightly higher in the horse populations studied (Table S1). 

The Jinjiang horse population showed medium values of the ROH parameters compared with other Chinese indigenous horse breeds. Although only a moderate degree of inbreeding occurred in the Jinjiang horse population in the past, based on the estimated value of F_ROH_, most of the individuals in the population possess the ROHs, and the inbreeding coefficient (f) of the breed indicated that Jinjiang horses currently may have the highest inbreeding level among the Chinese horse breeds studied.

Compared with the Chinese indigenous horse breeds, the foreign breeds, which have been subjected to highly selective breeding, such as Thoroughbreds, Andalusian horses, Arabian horses, Morgans, Shetland ponies, and Akhal_Teke horses, have higher N_ROH_ and F_ROH_. The results are consistent to the breeding history of these horse breeds.

### 3.4. Identical by Descent Analyses of the Jinjiang Horse

**Figure 4:**
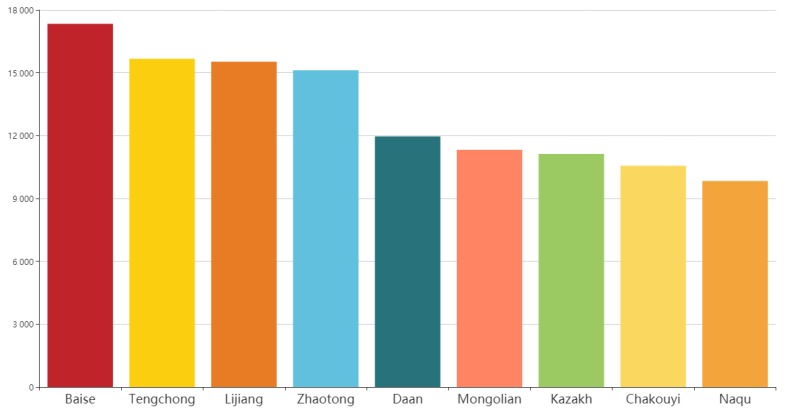
The estimation of Identity by Descent shared between the Jinjiang horse and other Chinese indigenous horse breeds.

The results of IBD analysis showed that Jinjiang horses are closely related to horse breeds in Southwest China (Figure 4). The Baise horse showed a significantly higher shared IBD with the Jinjiang horse compared to other Chinese breeds. The Naqu horse has the smallest IBD segment length shared with the Jinjiang horse. 

### 3.5. Identifying Signatures of Selection in the Jinjiang Horse Population with Runs of Homozygosity Analysis 

**Figure 5:**
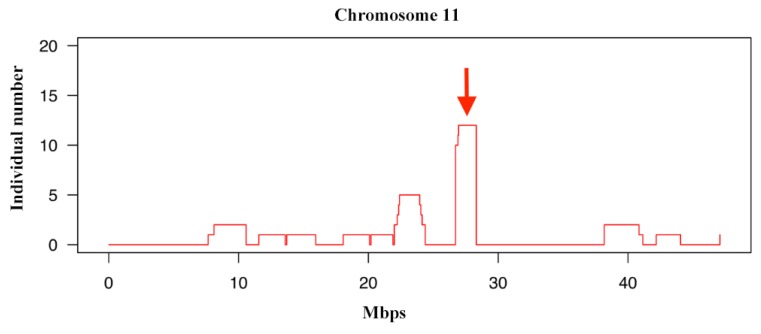
Visualization of runs of homozygosities (ROHs) per individual on chromosome 11.

Long consecutive homozygous genotype segments, i.e. ROHs, result when parents transmit identical haplotypes, which can be used for estimating autozygosity. Thus, we used PLINK software to compute the number of ROHs. Through investigating the distribution of ROHs across the genome, we detected the highest number of ROHs on ECA 11 of Jinjiang horses (Figure 5). An overview of the known genes in these regions (analyzed at the DAVID website: https://david.ncifcrf.gov/) is provided in Table 2. Among the genes, SPAG9 [38,39,40], NME1 [41,42], and NME2 [43,44] are involved in regulating the proliferation of cancer cells. MBDT1 is a gene regulating the development of embryonic skeletal system [45], UTP18 is a small subunit processome component [46], and CA10 is a zinc enzyme [47]. 

### 3.6. Estimation of Possible Ancestry with a Formal Test of ADMIXTURE 

Although above studies suggest that the Jinjiang horse has a diverse native-Chinese origin, the possible ancestry of Jinjiang Horses still remains unknown. Therefore, the f4 Ratio Estimation algorithm (f4 ratio = f4 (A, O; X, C)/f4 (A, O; B, C)) from ADMIXTOOLS Software Package [34] were used to conduct the further analysis. Baise horses (C) and Naqu horses (A) served as reference populations, and the Jinjiang horse is the target population (X) in f4 Ratio Estimation. In the output of the analysis, the results are positive only when the value of f4 ratio is above zero, and the greater the f4 ratio, the more likely it is that the B breed and Baise horses are the ancestors of Jinjiang horses. The results showed that Baise horses and Chakouyi horses are the most likely ancestors of Jinjiang horses (Table 3). 

### 3.7. Estimating the Proportion of Admixture

ALDER v1.03 software [35] was applied to estimate the proportions of Baise horse and Chakouyi horse in the ancestral sources of the Jinjiang horse. The Alder analysis, which is based on the exponential decay rate of admixture LD, was used to estimate the proportion of the gene flows between Jinjiang horses and the ancestral breeds. Our results showed that the proportion of Baise horses in the ancestry of Jinjiang horses is approximately 47%, and the Chakouyi horses account for approximately 20% of the ancestral sources.

## 4. Discussion

### 4.1. The Relationship between Jinjiang Horses and Worldwide Horse Populations

The earliest records about horse raising in Jinjiang of Fujian Province was in the Tang dynasty (AD 618-AD 907), which was much later than that of North China, where the remains of the earliest domestic horses in China were found. It was also later than the most horse-raising areas of Southwest China. It is unclear from where the early horses in Jinjiang were introduced. Because Quanzhou city was the most important port in the Marine Silk Road and there were frequent and prosperous trading activities between Quanzhou and the Arab world [48], it was hypothesized that some foreign horses, especially the horses from ancient Arabian countries, might contribute to the ancestral sources of Jinjiang horses [49]. Thus, it is essential to investigate the genetic links between Jinjiang horses and the main foreign horse breeds. 

To study the relationship of the Jinjiang horses and the worldwide horse populations, we created a merged dataset of 580 Chinese and foreign horses from 10 CHBs and 14 foreign horse breeds with 39,187 autosome SNPs, which were used to construct phylogenetic NJ trees (five donkeys used as outgroup). It was indicated that the Jinjiang horses have close relationship with other CHBs but did not show direct genetic link to any foreign horse breeds. Admixture analysis results also showed that the Jinjiang horse has the similar ancestral background to other CHBs, which is obviously different from foreign horse breeds. PCA and TreeMix analyses further confirmed the results of the clustering analysis and showed the close genetic relationship between Jinjiang horses and the other Chinese indigenous breeds, and there was no gene flow between Jinjiang horse and foreign horse breeds. These results showed that there is no significant genetic association between Jinjiang horses and foreign horses, and the Jinjiang horse was not influenced by the foreign breeds in history. Some of our results also showed that there is a close genetic relationship between European heavy horses and northern Chinese horses, especially with Daan horses, which was also confirmed in the TreeMix analysis. These phylogenetic results are in accordance with the breeding history of Daan horses, which had been bred by hybridizing the mares from local breeds with the sires of European heavy horses [50]. The genetic structure of the northern Chinese horses is similar to the Tuva horses and Caspian horses, which suggests the possible genetic links between horse populations of north China and the breeds in Eurasian steppes and supports the conclusion of our previous studies [11]. We also found evidence of genetic introgression from Arabian breed to Caspian horse, which is also consistent to the breeding records of Caspian horses [51]. The analysis also revealed known gene flows between horse breeds, such as Thoroughbreds and Morgan horses [25]. In summary, Jinjiang horses have a close relationship with other CHBs, and they are not closely related to foreign horses, including Arabian horses.

### 4.2. Native Origin of Jinjiang Horses

Because Jinjiang horses are only closely related to Chinese indigenous horses, we focused on the Chinese indigenous horses. Thus, the dataset of the 10 Chinese horse breeds (*n* = 234 and 57,597 SNPs) was involved in the subsequent analyses. The phylogenetic NJ tree (plus five donkeys as an outgroup) showed that most of the Jinjiang horses have a close relationship with the horse breeds in Southwest China, while a few individuals of the breed are relevant to the northern horses, which is attributed to the low selection imposed on the indigenous breed. Some stallions, which have no significant blood relationship, were used in breeding activities and were the founders of the subpopulations. These results are also in accord with the output of the LD analysis. The lowest LD level of Jinjiang horses suggested that Jinjiang horses did not experience intensive selection and that the genetic components of their ancestral sources may be largely preserved. The PCA and Admixture analysis confirmed the results of the phylogenetic NJ tree about the close genetic links between Jinjiang horses and the southwestern horse populations. The results of estimating genome-wide IBD sharing indicated that Jinjiang horses are most closely related to Baise horses among the southwest Chinese populations. Our study also revealed an evident admixture of Chakouyi horses and Jinjiang horses, suggesting a complicated history of gene flow from Chakouyi horses to Jinjiang horses. Further analysis revealed that Baise horses and Chakouyi horses are two of the most important ancestors of Jinjiang horses, and they account for approximately 47% and 20% of the ancestral sources of Jinjiang horses, respectively. Thus, the ancestry of Jinjiang horses is the mixture of Southwest and North Chinese horses. It is far away from the distributing areas of Baise horses (Baise of Guangxi Province) or Chakouyi horses (Tianzhu of Gansu Province) to those of Jinjiang horses (Jinjiang of Fijian Province)—i.e., over thousands of kilometers. The gene flows from Baise horse and Chakouyi horse were inevitably related with the historical trades and migrations of human beings, which may give reasonable explanation about the long-distance migrations of the horses. 

### 4.3. The Migrating Routes of the Ancestral Horses

The ancient Tea-Horse Road and Marine Silk Road in China may have a significant impact on the formation of the Jinjiang horse. From the Tang Dynasty (AD 618—AD 907) to the Qing Dynasty (AD 1636—AD 1912), the ancient Tea-Horse Road traversed through Yunnan province and Sichuan province to Tibet, which primarily aimed to trade tea from the tea-producing areas for horses from Tibet and other regions of horse-raising, but eventually it developed into arteries for trade in the mountainous regions of Southwest China, not only limited to Tea-Horse trading activities. In the mountainous ancient Tea-Horse Road, packhorses were used to cross difficult terrain, and horse-packing was the main means of transport for goods, while the businessmen who managed the horse-packing trade were called horse caravans. Guangxi Province, where high-quality tea is also produced, is at the eastern end of the ancient Tea-Horse Road. The ancient Heng-shan-zhai city of Guangxi Province, adjacent to Baise, had been an important and prosperous trade center in the Song Dynasty (AD 960–AD 1297). The ancient trade and the Tea-Horse Road facilitated the migrations and exchanges of horses in these areas [52]. The results of our study also confirmed the impact of the trade on the horse populations in Southwest China, which showed there are close genetic links among the Baise horse, the Tengchong horse, the Lijiang horse, and even the Naqu horse.

The Baise horse is an important horse breed in Guangxi Province. Because Guangxi Province was a major transportation and trade area (especially in the Song Dynasty), which connected inland provinces (such as Yunnan Province and Guizhou Province) to the coastal provinces, including Guangdong and Fujian where important ancient ports were located, the Baise horse had a profound influence on other horse breeds in the whole southwest region [53]. The Marine Silk Road was formed in the Tang Dynasty and bloomed in the Song Dynasty, starting from the southeastern coastal cities, Quanzhou (overlapping with the region where Jinjiang horses were distributed) in Fujian Province and Guangzhou in Guangdong Province, and passing through the sea route to West Asia and the Arab world. As the most prosperous port in the world at that time, Quanzhou attracted immigrating human beings and was the core city for the trading commodities of the surrounding areas. Quanzhou had established close ties with Guangxi Province through Guangdong Province [54]. In this way, the ancient Tea-Horse Road and the Marine Silk Road were also linked together through Guangdong Province and formed the passage through which the Baise horse and the Chakouyi horse migrated to Fujian Province.

Chakouyi horses are mainly distributed in the Gansu Province. The province is at the northern end of the Tibet-Yi Corridor, which represented the long routes (over 1000 km) along the north-south oriented rivers and valleys in the boundary of the southwest and northwest provinces of China. By following the corridor, people could migrate from the northwestern provinces to Southwest China, including Yunnan Province, Sichuan Province, and Tibet, all of which are areas along the ancient Tea-Horse Road [55]. Ancient people and their horses may have spread into the southern provinces through the Tibet-Yi Corridor and the ancient Tea-Horse Road, eventually affecting the indigenous horses in Fujian Province.

**Figure 6:**
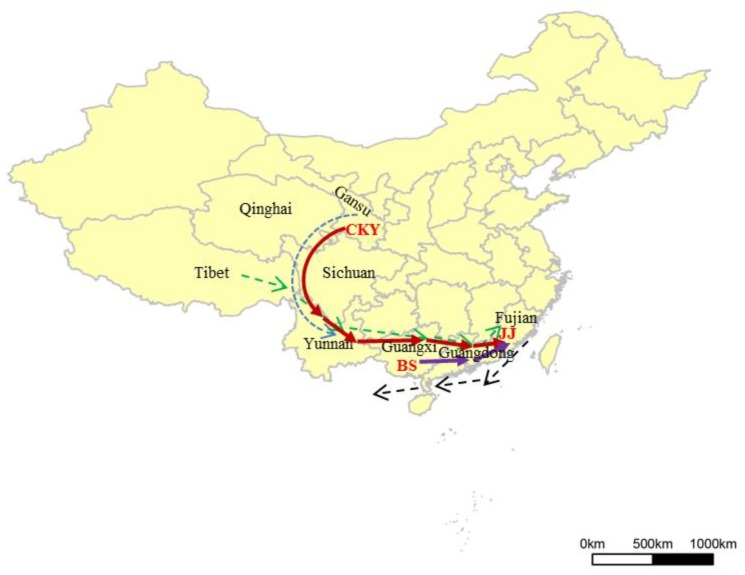
The diagram for the migration routes of the ancestral sources of Jinjiang horses. The broken blue lines indicate the Tibetan-Yi Corridor; the broken green lines, the Ancient Tea-Horse Road; the broken black lines, the Marine Silk Road (all of these ancient roads or corridors were simplified); the red heavy lines, the migrating routes of the Chakouyi horses; the purple heavy lines, the routes of the Baise horses; the black text, the names of the provinces; and the red text, the names of the horse breeds. (The map was figured with R Packages).

The hypothesized routes by which Baise horses and Chakouyi horses migrated to Fujian Province are shown below (Figure 6). Therefore, the formation of the Jinjiang Horse was closely related to ancient trading activities and the migration of human beings. 

### 4.4. The Conservation of Jinjiang Horses

After the population of Jinjiang horses continuously decreased in the past several decades, Jinjiang horses have become an endangered horse breed with a small population size and the conservation of the breed is now a pressing matter to carry out. 

From this study, the population structure and the genetic relationship of Jinjiang horses were elucidated, and the high level of inbreeding was also revealed with the analysis of ROH and the genetic diversity. The subpopulations of the breed could be used as main lines for further breeding programs and conservation. Moreover, the results regarding the genetic relationship among the individuals of the breeds also provide useful information for establishing sound mating systems for the population, thereby avoiding further inbreeding depressions. The ancestry sources revealed in the study can also be introduced to rejuvenate the Jinjiang horse. Although obvious association between the function of genes in the selected segments and the specific traits of Jinjiang horses was not found, the results provide valuable information for further studies to illustrate the signatures of selection, which offer meaningful information for the conservation of the breed. 

## 5. Conclusions

The results of our study suggest that Jinjiang horses originated from other Chinese indigenous breeds. The Baise horse and Chakouyi horse are two of the main ancestral sources of the Jinjiang horse. Ancient trading activities and the migration of human beings had deep impacts on the formation of the coastal horse breed.

The Jinjiang horse has been bred from their ancestors and is adapted to coastal southeast China, and it is essential to take action to conserve the indigenous breed and develop a new breeding strategy to improve the equestrian traits of the breed, through which it can eventually be developed into a high-quality saddle horse breed.

## Figures and Tables

**Table 1:** Parameters of ROH and Genetic Diversity of the worldwide horse studied breeds.

Breed	N	N_ROH_	S_ROH_	L_ROH_	F_ROH_	H_O_	H_E_	f
Baise	9	2.3	32,000.9	9850.0	0.0143	0.2818	0.3031	0.1024
Chakouyi	18	1.8	13,500.2	7574.4	0.0060	0.2824	0.3032	0.0397
Daan	17	2.9	29,691.3	9136.1	0.0132	0.2872	0.3064	0.0702
Jinjiang	19	5.8	74,697.5	9550.1	0.0333	0.2942	0.3102	0.0970
Kazakh	7	3.1	63,988.7	14,983.1	0.0285	0.2828	0.3041	0.0768
Lijiang	8	6.0	104,407.2	15,405.5	0.0465	0.2853	0.3067	0.1024
Inner Mongolian	10	4.4	69,589.7	9846.7	0.0310	0.2891	0.3078	0.0500
Naqu	14	1.9	14,013.9	6859.1	0.0062	0.2935	0.3092	0.0658
Tengchong	12	10.5	196,409.9	15,743.5	0.0876	0.2798	0.3105	0.0923
Zhaotong	21	7.9	112,960.4	12,476.9	0.0504	0.2802	0.3089	0.1446
Akhal_Teke	20	10.7	106,929.8	9919.2	0.0477	0.2748	0.3082	0.0499
Andalusian	18	16.9	229,341.4	12,841.3	0.1022	0.2810	0.3102	0.1182
Arabian	24	14.0	156,306.3	10,144.7	0.0697	0.2829	0.3115	0.0857
Belgian	30	11.7	116,769.2	9753.1	0.0521	0.2928	0.3116	0.1566
Caspian	11	5.2	47,479.8	8895.2	0.0212	0.2864	0.3081	0.0210
Finnhorse	27	5.3	59,737.2	11,224.1	0.0266	0.2879	0.3064	0.1001
Hanoverian	15	9.5	82,050.0	8402.7	0.0366	0.2872	0.3078	−0.0433
Mongolian	3	3.3	29,864.8	8312.9	0.0133	0.2891	0.3078	0.0793
Morgan	43	13.1	167,297.9	11,061.9	0.0746	0.2955	0.3088	0.0780
Percheron	20	7.1	63,734.8	8336.1	0.0284	0.2871	0.3082	0.1212
Quarter	40	9.3	88,200.5	9245.7	0.0393	0.2911	0.3090	−0.0260
Shetland	27	17.5	185,601.4	10,109.3	0.0827	0.2844	0.3054	0.2434
Thoroughbred	36	23.6	222,096.8	9489.8	0.0990	0.2875	0.3059	−0.0019
Tuva	11	4.0	58,334.8	10,996.3	0.0260	0.2851	0.3088	0.0675

Note: ROHs, runs of homozygosity segments; N, number of samples having ROH; N_ROH_, number of ROH; S_ROH_, genome length covered by ROH; L_ROH_, average of ROH; F_ROH_, autozygosity of ROH; H_O_, observed heterozygosity; H_E_, expected heterozygosity; f, inbreeding coefficient; Baise, Baise horses; Chakouyi, Chakouyi horses; Daan, Daan horses; Jinjiang, Jinjiang horses; Kazakh, Kazakh horses; Lijiang, Lijiang horses; Inner Mongolian, Inner Mongolian horses; Naqu, Naqu horses; Tengchong, Tengchong horses; Zhaotong, Zhaotong horses.

**Table 2:** Runs of homozygosity islands shared by most individuals in Jinjiang horses.

Gene Stable ID	Chr.	Begin	End	Know Genes
ENSECAG00000015256	11	26,497,130	26,746,723	SPAG9
ENSECAG00000028963	11	26,767,419	26,776,603	NME1
ENSECAG00000021658	11	26,779,860	26,782,544	NME2
ENSECAG00000022700	11	26,787,953	26,824,364	MBTD1
ENSECAG00000008260	11	26,852,773	26,888,439	UTP18
ENSECAG00000013061	11	27,143,031	27,609,842	CA10

**Table 3:** Estimation of the genome-wide possible ancestry of Jinjiang horses.

A	O	X	C	A	O	B	C	F4 Ratio	Std.err	Z (null = 0)
NQ	Donkey	JJ	BS	NQ	Donkey	CKY	BS	39.2431	12.654	3.1019d
NQ	Donkey	JJ	BS	NQ	Donkey	DA	BS	−0.0990	0.2196	−0.4519d
NQ	Donkey	JJ	BS	NQ	Donkey	KZK	BS	−0.0674	0.5070	−0.1339d
NQ	Donkey	JJ	BS	NQ	Donkey	LJ	BS	−0.6912	1.2101	−0.5719d
NQ	Donkey	JJ	BS	NQ	Donkey	IMG	BS	−0.1099	0.3299	−0.3339d
NQ	Donkey	JJ	BS	NQ	Donkey	TC	BS	−5.3502	3.0578	−1.7509d
NQ	Donkey	JJ	BS	NQ	Donkey	ZT	BS	0.3187	0.4074	0.7829d

Note: A, the least likely ancestor of Jinjiang horses. The Naqu horse (NQ) was selected as A, according to the IBD results. O, Outgroup, donkey; X, target breed, Jinjiang Horse (JJ); C, the most likely ancestor of Jinjiang horses. The Baise horse (BS) was set as C according to the IBD results. B, a possible ancestor to be tested, including the Chakouyi horse (CKY), the Daan horse (DA), the Kazakh horse (KZK), the Lijiang horses (LJ), the Inner Mongolian horse (IMG), Tengchong horse (TC), and the Zhaotong horse (ZT).

**Figure A1:**
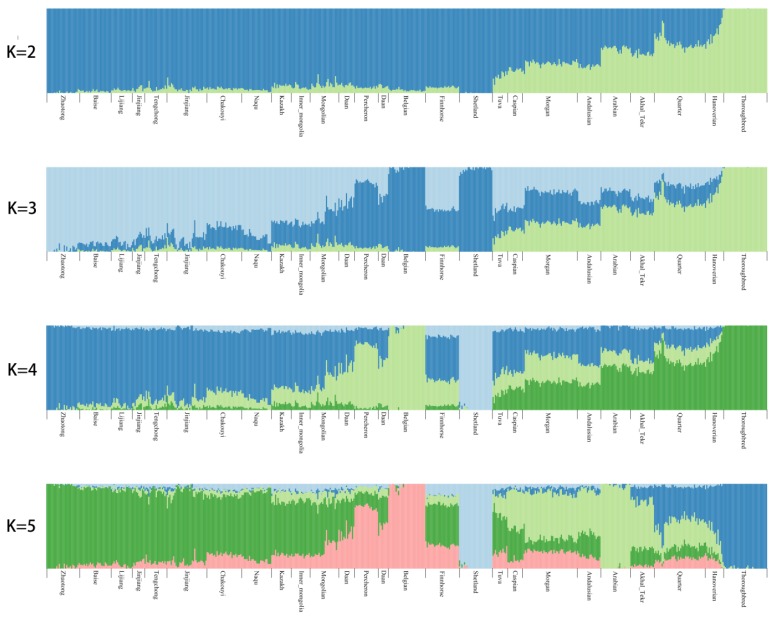

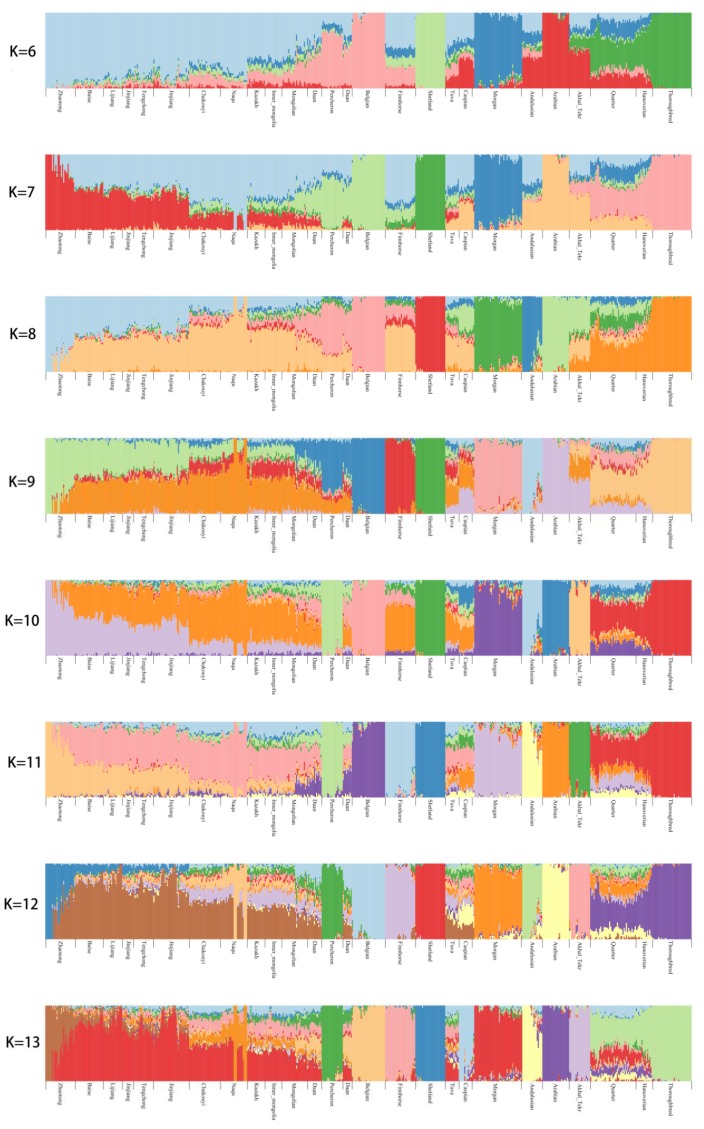

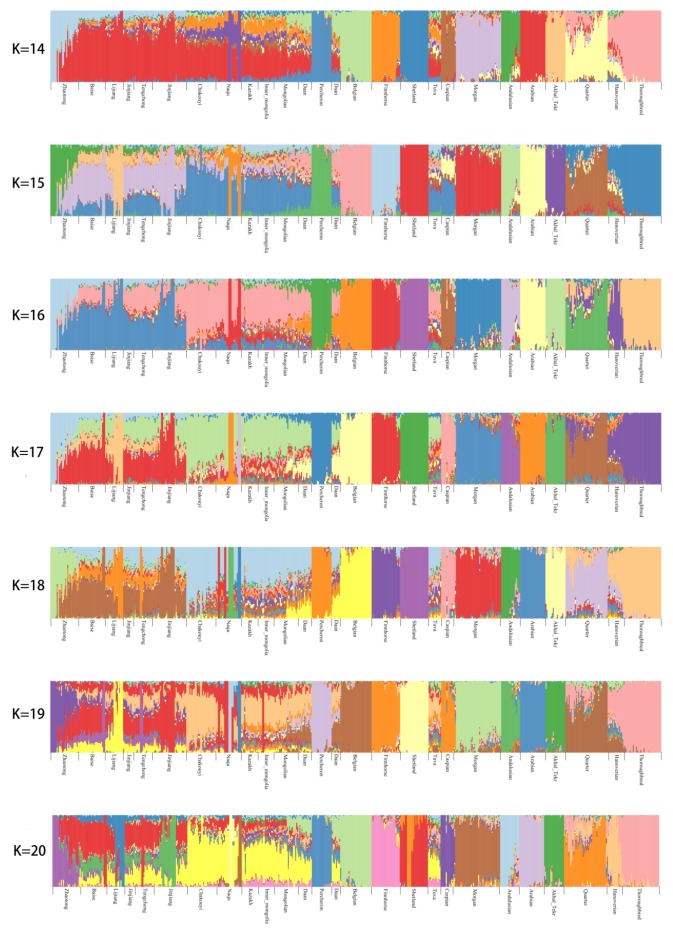
Analysis of population structure on both foreign and Chinese horses (K = 2–20).

**Figure A2:**
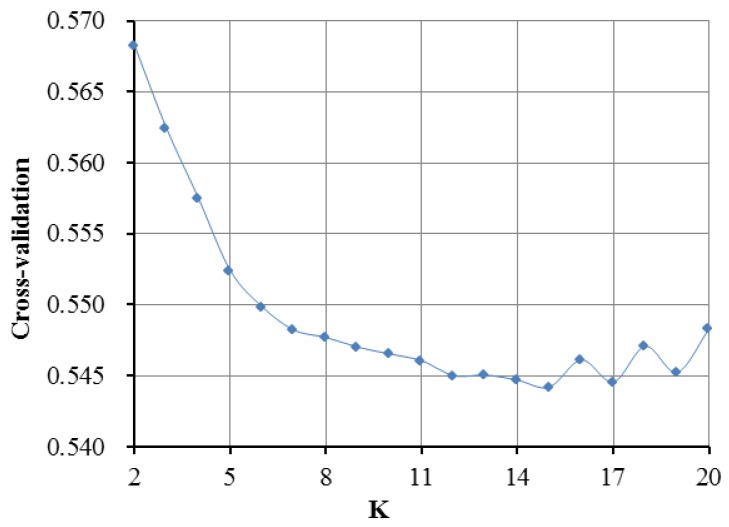
Cross-validation for each run in Admixture analysis for both foreign and Chinese horses.

**Figure A3:**
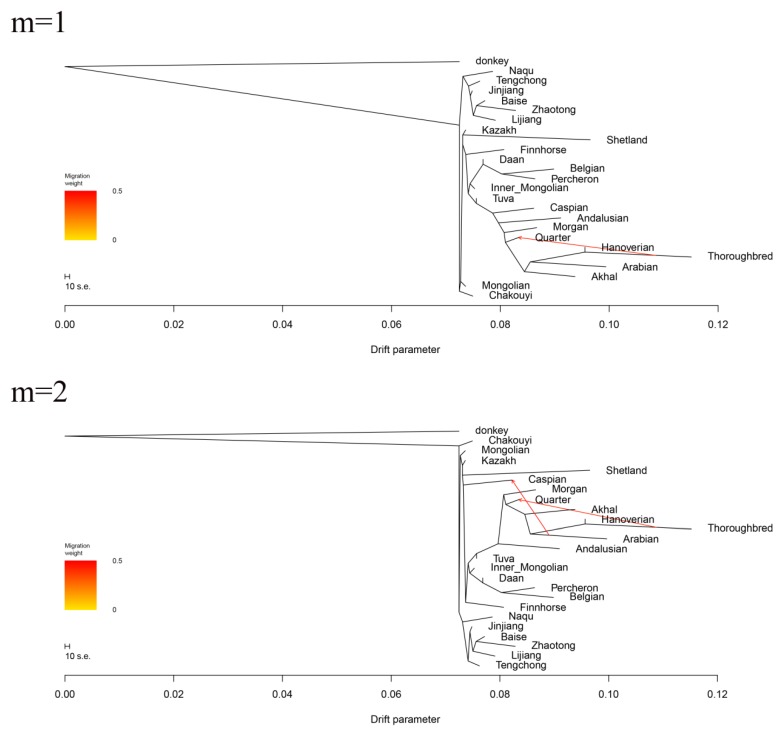

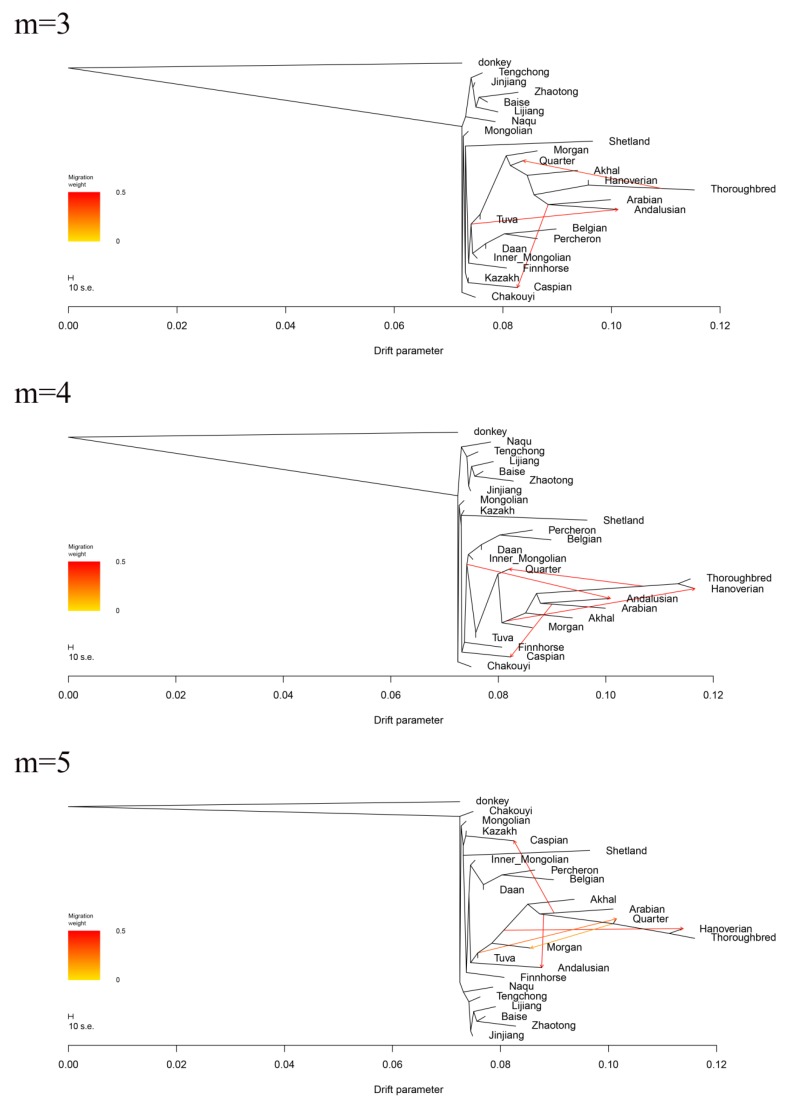

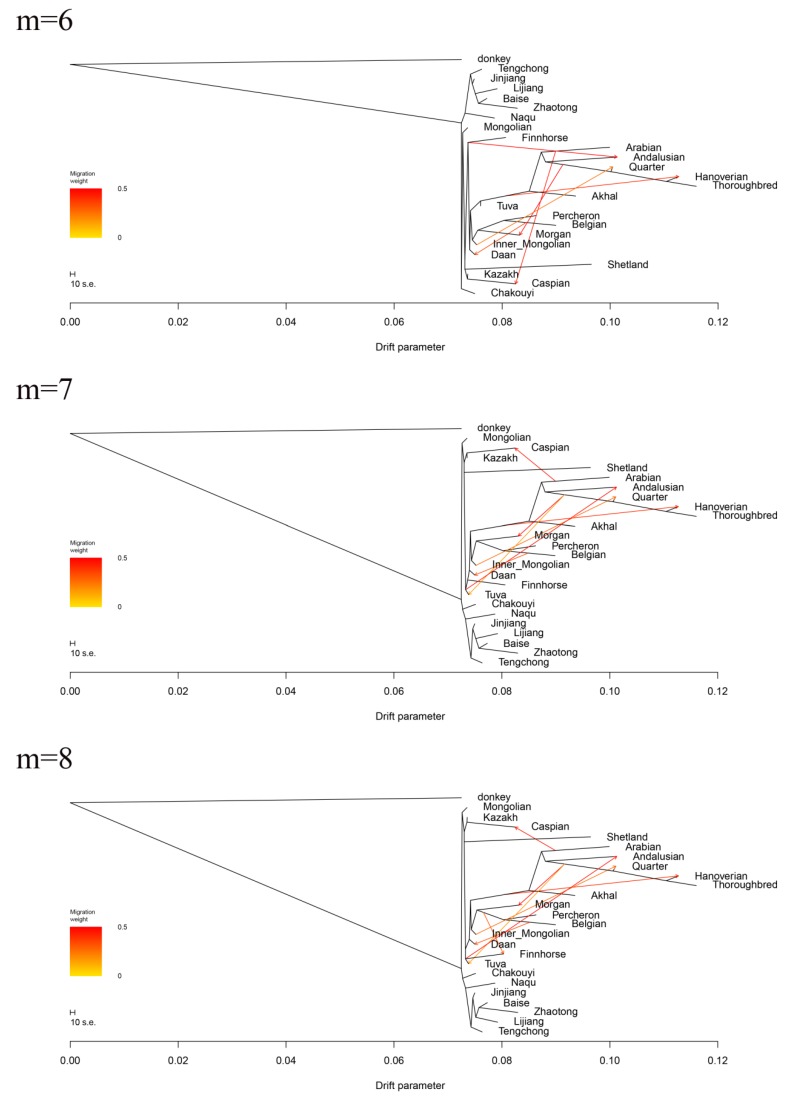
Phylogenetic network of the inferred relationships among foreign and Chinese horses.

**Figure A4:**
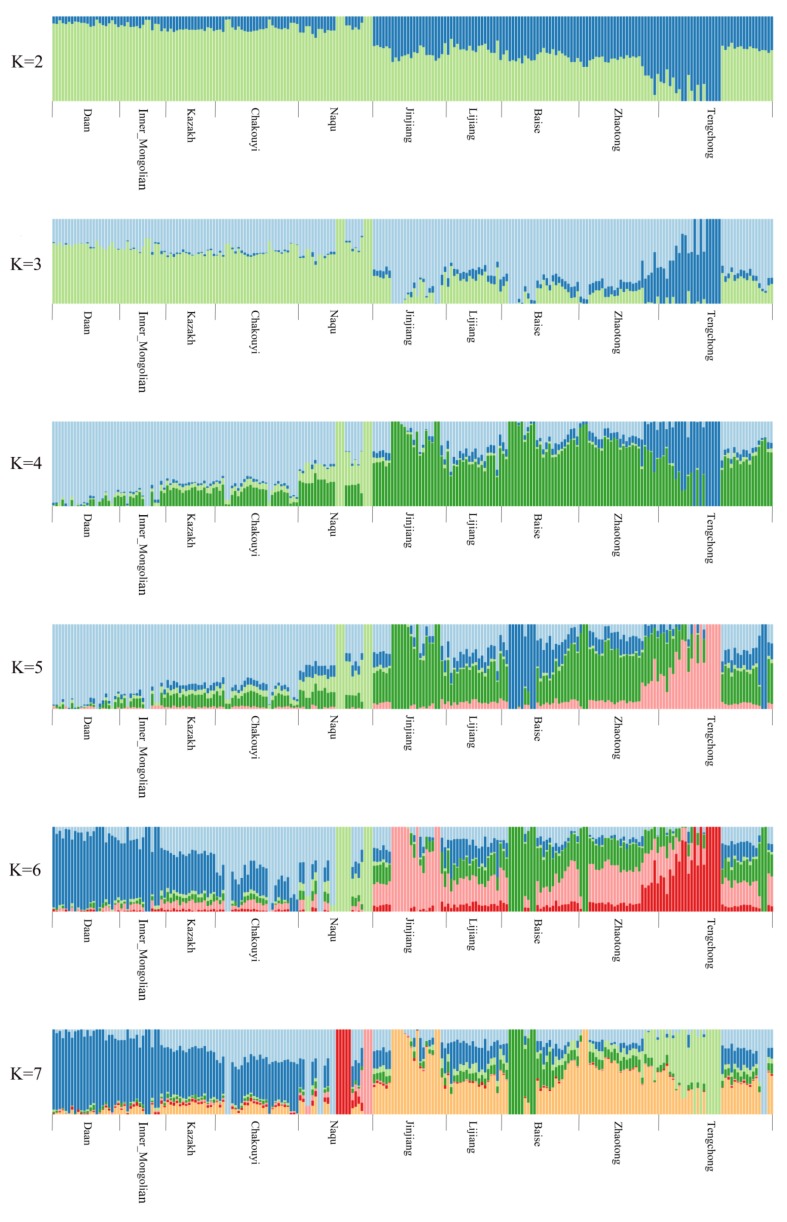

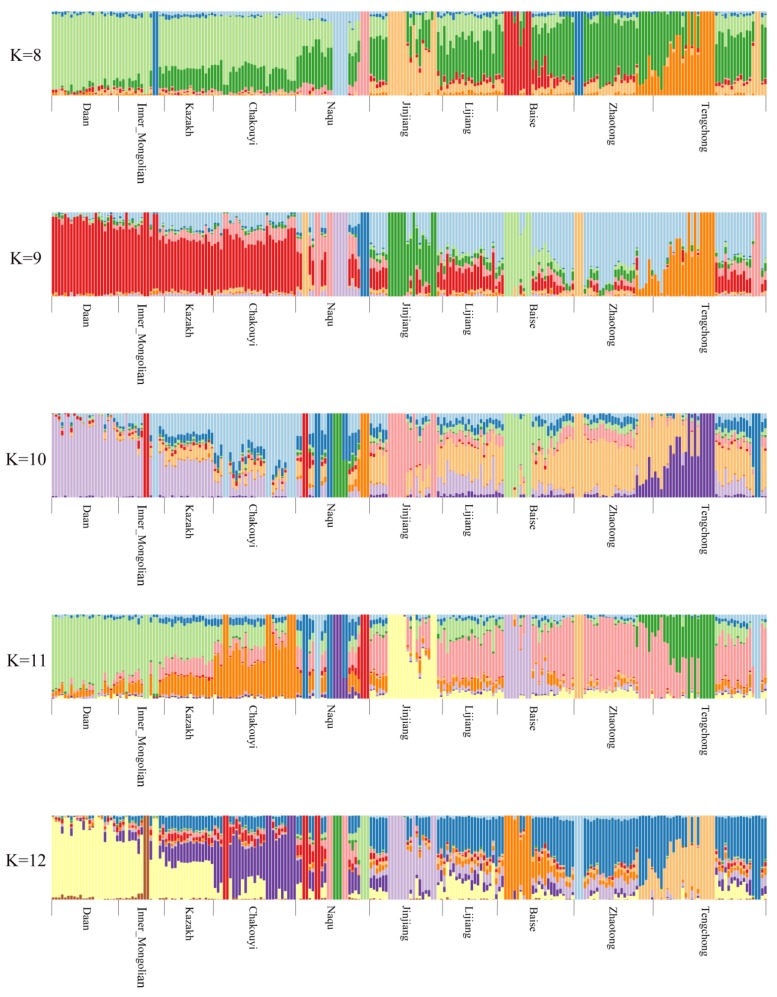
Analysis of the population structure of Chinese horses (K = 2–12).

**Figure A5:**
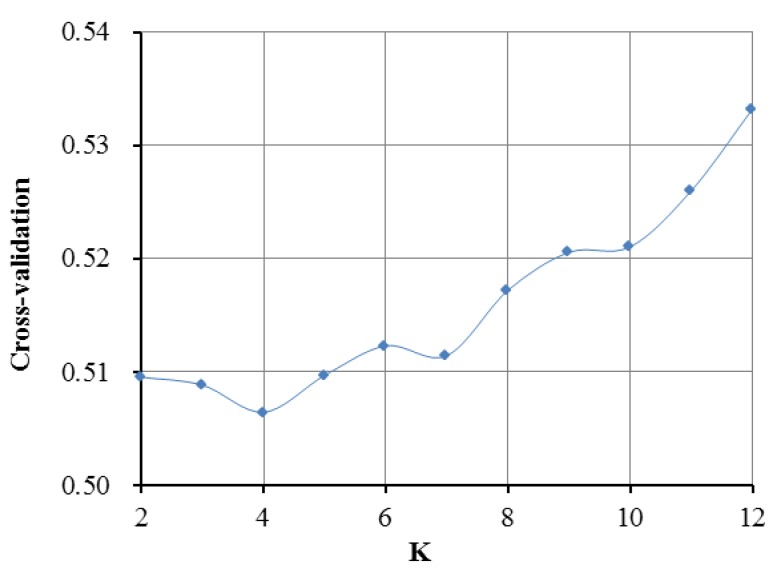
Cross-validation for each run in Admixture analysis of Chinese horses.

**Figure A6:**
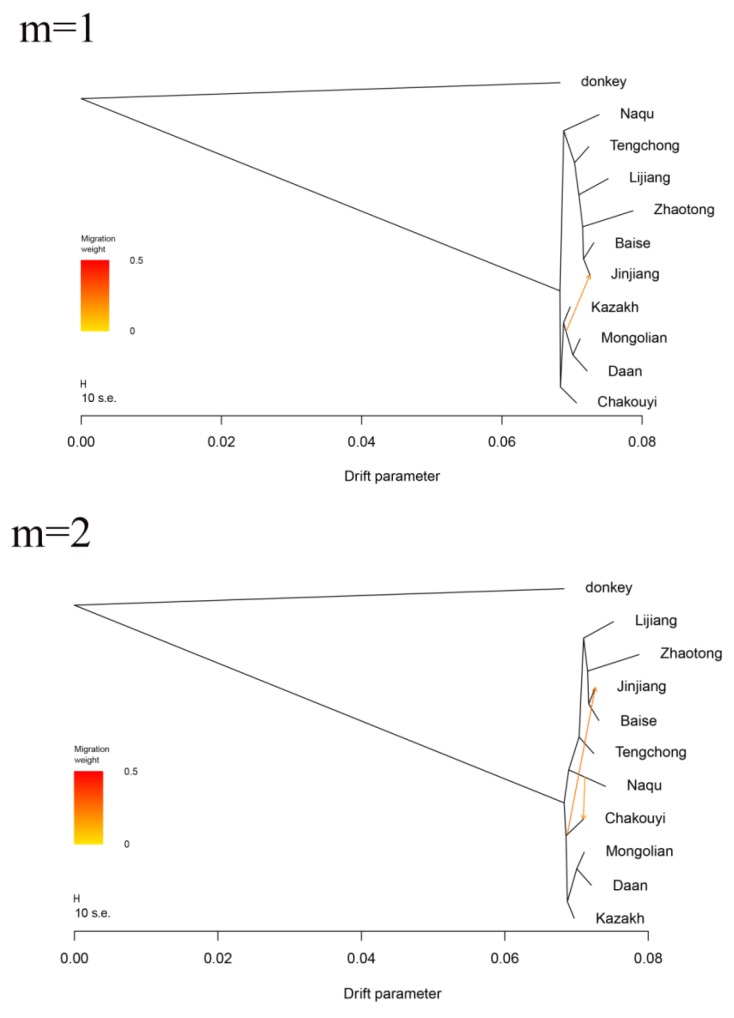

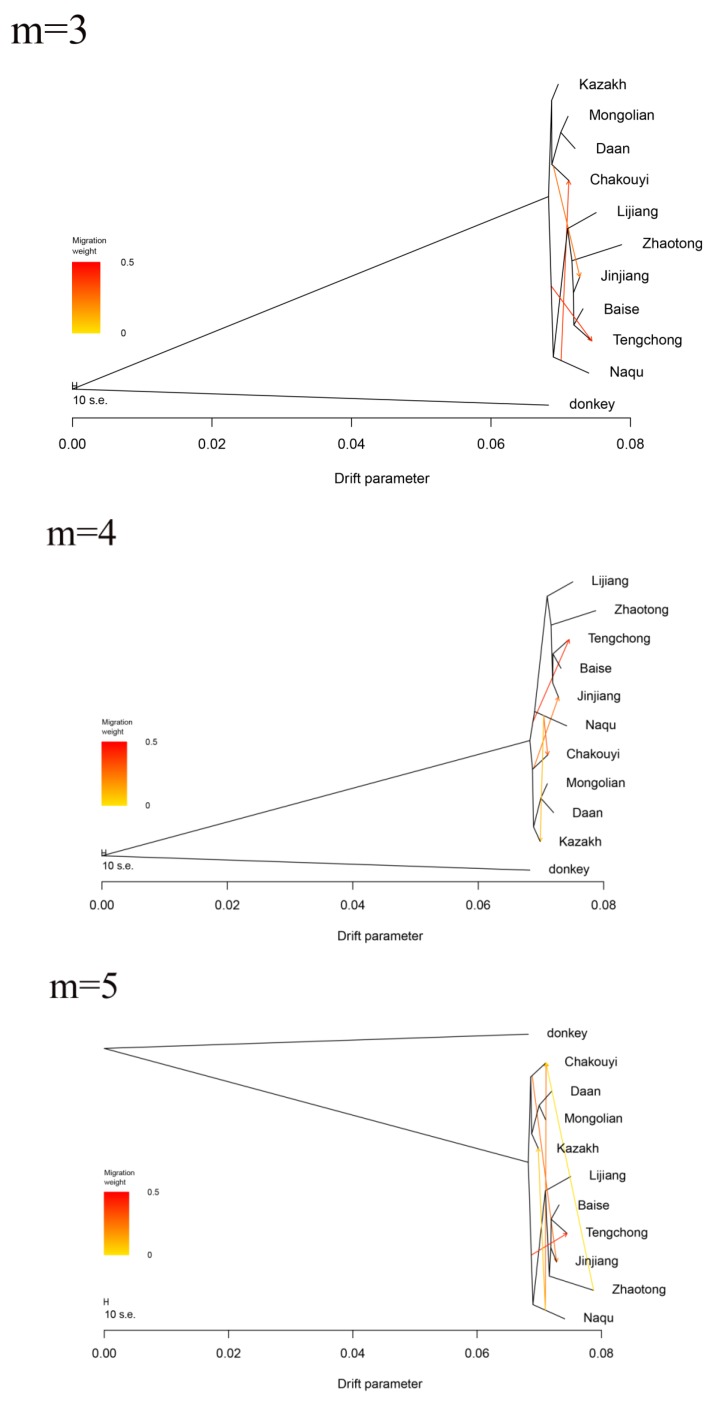
Phylogenetic network of the inferred relationships among 10 Chinese indigenous horse breeds.

## Supplementary Materials

The following are available online at https://www.mdpi.com/2073-4425/10/3/241/s1. Table S1: Sample information of 24 worldwide horse breeds, Table S2: Information for all Chinese indigenous horse breeds.
