# Supplementary material for: The Origin of a Coastal Indigenous Horse Breed in China Revealed by Genome-Wide SNP Data
Source: Genes (Basel). 2019 Mar 21;10(3):241. doi: 10.3390/genes10030241 (PMC6471023; doi:10.3390/genes10030241)
Supplement: Supplementary file 1 [file genes-10-00241-s001.zip › Table S1.docx]

Table S1: Sample information of 24 horse breeds.

| **Breed Name** | **Original horse Number** | **Horse number After quality control** | **Country** | **Region** | **Origin** |
| --- | --- | --- | --- | --- | --- |
| Kazakh | 17 | 16 | China | East_Asia | This Study |
| Inner_Mongolian | 23 | 15 | China | East_Asia | This Study |
| Daan | 26 | 22 | China | East_Asia | This Study |
| Chakouyi | 34 | 28 | China | East_Asia | This Study |
| Naqu | 29 | 24 | China | East_Asia | This Study |
| Jinjiang | 57 | 44 | China | East_Asia | This Study |
| Zhaotong | 26 | 26 | China | East_Asia | This Study |
| Tengchong | 22 | 17 | China | East_Asia | This Study |
| Lijiang | 31 | 18 | China | East_Asia | This Study |
| Baise | 36 | 25 | China | East_Asia | This Study |
| Mongolian | 18 | 18 | Mongolian | East_Asia | Petersen et al., 2013 |
| Tuva | 15 | 15 | Russian | East_Asia | Petersen et al., 2013 |
| Arabian | 24 | 24 | Arabian | West_Asia | Petersen et al., 2013 |
| Caspian | 16 | 14 | Iran | West_Asia | Petersen et al., 2013 |
| Akhal_Teke | 20 | 19 | Turkmenistan | Central_Asia | Petersen et al., 2013 |
| Finnhorse | 27 | 27 | Finland | Northern_Europe | Petersen et al., 2013 |
| Andalusian | 18 | 18 | Spain | Southern_Europe | Petersen et al., 2013 |
| Hanoverian | 15 | 15 | Germany | Central_Europe | Petersen et al., 2013 |
| Belgian | 30 | 30 | Belgium | West_Europe | Petersen et al., 2013 |
| Shetland | 27 | 27 | England | West_Europe | Petersen et al., 2013 |
| Throughbred | 36 | 36 | England | West_Europe | Petersen et al., 2013 |
| Percheron | 23 | 19 | France | West_Europe | Petersen et al., 2013 |
| Morgan | 43 | 43 | America | America | Petersen et al., 2013 |
| Quarter_Horse | 40 | 40 | America | America | Petersen et al., 2013 |
| Dezhou Donkey | 5 | 5 | China | East_Asia | This Study |
