# Supplementary material for: The Origin of a Coastal Indigenous Horse Breed in China Revealed by Genome-Wide SNP Data
Source: Genes (Basel). 2019 Mar 21;10(3):241. doi: 10.3390/genes10030241 (PMC6471023; doi:10.3390/genes10030241)
Supplement: Supplementary file 1 [file genes-10-00241-s001.zip › Table S2.docx]

Table S2: Sample information of 10 Chinese indigenous horse breeds.

| **Breed Name** | **Original horse Number** | **The horse number after qulity control** | **Country** | **Group** | **Origin** |
| --- | --- | --- | --- | --- | --- |
| Kazakh | 17 | 16 | China | Kazakh Horse Tupe | This Study |
| Inner_Mongolian | 23 | 15 | China | Mongolian Horse Type | This Study |
| Daan | 26 | 22 | China | Mongolian Horse Type | This Study |
| Chakouyi | 34 | 27 | China | Hequ Horse Type | This Study |
| Naqu | 29 | 24 | China | Tibetan Horse Type | This Study |
| Jinjiang | 57 | 44 | China | Southwest Horse Type | This Study |
| Zhaotong | 26 | 26 | China | Southwest Horse Type | This Study |
| Tengchong | 22 | 17 | China | Southwest Horse Type | This Study |
| Lijiang | 31 | 18 | China | Southwest Horse Type | This Study |
| Baise | 36 | 25 | China | Southwest Horse Type | This Study |
| Dezhou Donkey | 5 | 5 | China | East_Asia | This Study |
